# Cross-species genomic and functional analyses identify a combination therapy using a CHK1 inhibitor and a ribonucleotide reductase inhibitor to treat triple-negative breast cancer

**DOI:** 10.1186/bcr3230

**Published:** 2012-07-19

**Authors:** Christina N Bennett, Christine C Tomlinson, Aleksandra M Michalowski, Isabel M Chu, Dror Luger, Lara R Mittereder, Olga Aprelikova, James Shou, Helen Piwinica-Worms, Natasha J Caplen, Melinda G Hollingshead, Jeffrey E Green

**Affiliations:** 1 Laboratory of Cancer Biology and Genetics, National Cancer Institute, National Institutes of Health, 37 Convent Drive, Bethesda, MD 20892; 2 Department of Cell Biology and Physiology and Department of Internal Medicine, BRIGHT Institute, Washington University, 425 S. Euclid Avenue, St. Louis, MO 63110; 3 Genetics Branch, National Cancer Institute, National Institutes of Health, 37 Convent Drive, Bethesda, MD 20892; 4 Biological Testing Branch, National Cancer Institute, National Institutes of Health, 1050 Boyles Street, Frederick, MD 21702

## Abstract

**Introduction:**

Triple-negative breast cancer (TNBC) is an aggressive subtype of breast cancer that is diagnosed in approximately 15% of all human breast cancer (BrCa) patients. Currently, no targeted therapies exist for this subtype of BrCa and prognosis remains poor. Our laboratory has previously identified a proliferation/DNA repair/cell cycle gene signature (Tag signature) that is characteristic of human TNBC. We hypothesize that targeting the dysregulated biological networks in the Tag gene signature will lead to the identification of improved combination therapies for TNBC.

**Methods:**

Cross-species genomic analysis was used to identify human breast cancer cell lines that express the Tag signature. Knock-down of the up-regulated genes in the Tag signature by siRNA identified several genes that are critical for TNBC cell growth. Small molecule inhibitors to two of these genes were analyzed, alone and in combination, for their effects on cell proliferation, cell cycle, and apoptosis *in vitro *and tumor growth *in vivo*. Synergy between the two drugs was analyzed by the Chou-Talalay method.

**Results:**

A custom siRNA screen was used to identify targets within the Tag signature that are critical for growth of TNBC cells. Ribonucleotide reductase 1 and 2 (RRM1 and 2) and checkpoint kinase 1 (CHK1) were found to be critical targets for TNBC cell survival. Combination therapy, to simultaneously attenuate cell cycle checkpoint control through inhibition of CHK1 while inducing DNA damage with gemcitabine, improved therapeutic efficacy *in vitro *and in xenograft models of TNBC.

**Conclusions:**

This combination therapy may have translational value for patients with TNBC and improve therapeutic response for this aggressive form of breast cancer.

## Introduction

Triple negative breast cancer (TNBC) is an aggressive and heterogeneous subtype of breast cancer defined by the absence of estrogen (ER) and progesterone (PR) steroid hormone receptor expression and lacking high expression and/or amplification of HER2/ERBB2. Although TNBC represents only 10% to 15% of breast cancer diagnoses, it disproportionately affects pre-menopausal women and African-American women and is associated with poor prognosis [1]. Due to the absence of hormone receptor expression and lack of human epidermal growth factor receptor 2 **(**HER2) overexpression, no targeted therapies exist for TNBC, which limits treatment to standard chemotherapy [2]. Paradoxically, women with TNBC have a significantly higher rate of pathologic complete response (pCR) to standard chemotherapy compared to other types of breast cancer [3,4]. Yet those TNBC patients who do not undergo a pCR generally experience recurrence within the first three years and poor overall survival due to an increased incidence of distant node, lung, and brain metastases [5]. Thus, identification of drugs that target specific molecular features of TNBC and the use of improved preclinical models for this disease are important research priorities.

Mutations in p53 and loss of function of the pRb pathway are found in the majority of TNBCs. These mutations lead to the dysregulation of many genes, including genes that regulate the cell cycle and apoptosis, and may account for the particularly aggressive properties of this form of breast cancer [1]. More than 44% of TNBCs have been found to harbor p53 mutations [1], whereas loss of Rb function occurs in at least 70% of TNBCs [6,7]. In order to identify potential molecular targets for TNBC related to loss of the critical tumor suppressor functions of p53 and pRb, we hypothesized that identification of a gene expression signature based upon the expression of an oncoprotein whose mechanism of transformation results in the inhibition of p53 and Rb function would be highly relevant to human TNBC. We previously identified a common gene expression signature (Tag signature) comprised of approximately 120 named genes based upon the loss of p53 and Rb functions in several transgenic mouse models of epithelial cancers (including the C3(1)/Tag model of mammary cancer) where the functions of these two tumor suppressor genes are abrogated by the expression of the SV40 T-antigen (Tag) oncoprotein [8]. Tag is known to bind to and functionally inactivate both p53 and the pRb family of proteins, thus providing a means to simultaneously inhibit the tumor suppressor activities of these proteins. The molecular relevance of Tag-induced mammary cancer arising in the C3(1)/Tag model to human TNBC has been clearly demonstrated through gene expression profiling. It revealed that the C3(1)/Tag transgenic model is the genetically-engineered mouse model of mammary cancer most closely related to human TNBC [9] and shares many other important biological features of the human disease [8-10].

Further analyses revealed that the Tag signature is highly represented in human TNBC and could distinguish triple negative from other forms of breast cancer [8]. Contained within the Tag signature are genetic nodes related to the functions of p53, pRb, MYC, and genes regulating apoptosis [8]. The 120-gene signature contains genes involved in DNA metabolism and replication (*Dhfr, Top2a, Tyms*), DNA repair (*Claspin, Rrm1, Pola1*), chromosome maintenance *(Plk4, Mcm *genes), cell cycle regulation (*Pk2, Chk1*), cell replication and proliferation (*Cdc28, Ki67, Pcna*), microtubule stabilization (*Kif11, Stmn1*), and apoptosis (*Birc5, Casp2*), suggesting that the expression of genes contained within this signature could be vital for the survival and maintenance of this aggressive form of human breast cancer.

We hypothesized that some of the dysregulated genes contained in the Tag signature are essential for the survival of TNBC cells either alone or in combination. In order to test this hypothesis, the up-regulated genes within the Tag signature were knocked-down in human TNBC cells using a custom siRNA library. This screen identified the two subunits of ribonucleotide reductase, *RRM1 *and *RRM2*, and the checkpoint kinase *CHK1*, as particularly sensitive targets resulting in the reduced survival of TNBC cells. These results were further validated both *in vitro *and *in vivo *using gemcitabine, an inhibitor of RRM1 and RRM2, and UCN-01 and AZD 7762, inhibitors of CHK1, using several human triple negative cell lines and the C3(1)/Tag transgenic model of TNBC. Since CHK1 activation results in cell cycle arrest that is necessary for DNA repair, and RRM1 and RRM2 are critical for DNA synthesis and repair, we further hypothesized and demonstrated that inhibiting CHK1, RRM1 and RRM2 through combined treatment with gemcitabine and UCN-01 resulted in greater therapeutic efficacy than either agent alone. These results demonstrate that a gene signature identified through cross-species analysis of relevant molecular pathways can be useful for the identification of targets for TNBC.

## Materials and methods

### Reagents

For *in vitro *assays, therapeutic agents were purchased as noted. UCN-01 and gemcitabine hydrochloride, were purchased from Sigma-Aldrich (St. Louis, MO, USA). The specific CHK1 inhibitor, AZD 7762, was provided by the Helen Piwinica-Worms laboratory (Washington University in St. Louis, MO, USA) [11]. All therapeutic agents were dissolved in dimethyl sulfoxide (DMSO), aliquoted, and stored at -20°C.

### Cell culture and drug treatment

Human breast cancer cell lines MDA-MB-231, Hs578T, SUM 159, HCC1187, BT-549 and MCF-7 were purchased from the American Type Culture Collection (Manassas, VA, USA). The mouse mammary tumor cell line, M6, was derived from a C3(1)TAg mouse mammary tumor as reported previously [12]. Growth media for MDA-MB-231 cells (RPMI medium 1640) and M6 cells ((D)MEM (high glucose)) were supplemented with 5%(v/v) fetal bovine serum (FBS), penicillin-streptomycin and MEM sodium pyruvate (100 mM). Hs578T cells were cultured in (D)MEM high glucose supplemented with 10% FBS, penicillin-streptomycin and MEM sodium pyruvate solution 100 mM. SUM 159 cells were cultured in Ham's F-12 medium supplemented with 5% FBS, 5 μg/mL recombinant human insulin, 1 μg/mL hydrocortisone, and 10 mM HEPES. BT-549 cells were cultured with RPMI-1640 medium supplemented with 10% FBS and 0.852 μg/mL recombinant human insulin. HCC1187 cells were cultured in RPMI-1640 media supplemented with 10% FBS and penicillin-streptomycin. MCF-7 cells were cultured in (D)MEM (high glucose) supplemented with 10% FBS, penicillin-streptomycin, MEM sodium pyruvate solution 100 mM, and MEM non- essential amino acids. Normal human breast epithelial cells, M98040 and M99005, were provided by Ofelia Olivero (National Institutes of Health, Bethesda MD, USA) and cultured as reported [13]. MCF10A cells (M-I) were provided by Lalage Wakefield (National Institutes of Health) and cultured as described [14]. All cell culture reagents were purchased from Invitrogen (Gaithersburg, MD, USA).

### Microarray data

Four microarray data sets were analyzed. GEMII cDNA array data profiles of SV40T/t-antigen mouse mammary tumors and normal mammary tissue (FVB background), which were used to derive the SV40-Tag signature (Tag signature), are described elsewhere [8]. The human MDA-MB-231 breast cancer cell line and M98040/M99005 epithelial cells were profiled on HG-U133Plus2 chips, Affymetrix (Santa Clara, CA, USA) and processed with GC-RMA and quantile normalization [15]. The data for the M6 mouse breast cancer cell line, other C3(1)/Tag mouse mammary tumors and normal mammary tissue samples (FVB background) were generated using Affymetrix MOE430A arrays and processed with RMA and quantile normalization [15]. Publicly available data for 51 human breast cancer cell lines were obtained from Neve *et al*. [16]. The SV40 T/t-antigen-specific gene signature was mapped between platforms and/or species using Entrez Gene ID and JAX homology with the annotation provided in the mAdb database [17]. Multiple array probes mapping to the same Entrez IDs were reduced to single probes with the highest median signal across all the samples. Prior to integration of the four data sets each gene expression was standardized to the distribution of the mean of zero and unit standard deviation (z-score transformation). The analyses were performed with R statistical programming [18] and the Bioconductor *affy *and *gcrma *packages [19].

### siRNA library and screen

A custom-designed gene library of 108 pools of four siRNA oligos [see Additional file 1, Table S1] was used for the siRNA screen (siGenome, Dharmacon, Lafayette CO, USA). In addition to the siRNA library wells, each plate contained three replica wells with negative controls (Dharmacon) (pool of non-targeting siRNA (NTS) and cyclophilin B siRNA) and positive controls (*Plk1 *siRNA (Dharmacon) and All-Star Cell Death siRNA (Qiagen, Valencia, CA, USA). The Tag siRNA library consists of siRNA oligo pools, 4 oligos/pool, for each of the 80 up-regulated genes in the Tag signature. The library also contains 28 genes that have recently been associated with TNBC [20,21].

### siRNA transfections

Transfections were performed by pre-complexing siRNA (1 pmol) with Oligofectamine lipid transfection reagent (Invitrogen) in serum-free media (RPMI for MB-MDA-231, (D)MEM for HS578T or (D)MEMF12 for MCF10A ) in individual wells for 30 minutes at room temperature (RT). Cells were added in media supplemented with 2x FBS resulting in final concentrations of 10 nM siRNA and 5% FBS for MB-MDA-231 (50 nM and 10% FBS for HS578T and MCF10A). The cell/siRNA mix was incubated at RT for 45 minutes before being placed at 37 °C in a humidified atmosphere containing 5% CO2 for the times indicated. Primary siRNA library screens were performed in 96 well plates (utilizing the interior 60 wells only to reduce data variation as a result of evaporation at the edges of the plate), incubated for 72 hours and analyzed for proliferation changes. RNA, for quantitative real-time PCR, was isolated from 12 well plates 24 hours post-transfection. Protein studies, for immunoblot analysis, were performed in 6-well dishes and harvested 48 hours post-transfection.

The mean absorbance values per gene target per plate were normalized using the mean value for non-targeting siRNA (NTS) transfected cells. *Z*-*scores *were calculated relative to the plate mean and standard deviation.

For de-convolution studies, four siRNAs targeting a given gene were evaluated individually, each used at a concentration of 10 nM, and compared to non-targeted siRNA #2 (Dharmacon).

### Proliferation assay

Cells were counted and plated in 96-well cell culture plates. Twenty-four hours later cells were treated with therapeutic agents as described. At time points indicated, cell proliferation was assayed with CellTiter 96^R ^Aqueous Non-Radioactive Cell Proliferation Assay following manufacturers protocol (Promega, Madison, WI, USA). Data points represent an average of three samples per treatment and experiments were repeated at least three times.

### mRNA gene expression analysis

RNA was extracted from cells using TRIzol reagent (Invitrogen) and purified using the RNeasy Mini kit (Qiagen) according to the manufacturer's instructions. RT-PCR was performed on 500 ng RNA using TaqMan reverse transcription reagents (Applied Biosystems, Carlsbad, CA). Quantitative real time PCR was performed using iQSybr Green Supermix (BioRad, Hercules, CA,) and quantified using an iCycler Real Time PCR unit (BioRad) using *PPIA *as an internal control. The primers used for each gene are as follows: *RRM1 *(F: AAG AGC AGC GTG CCA GAG AT, R: ACA CAT CAA AGA CCA GTC CTG ATT AG) [22], *RRM2 *(F: GCA GCA AGC GAT GG CAT AGT, R: GGG CTT CTG TAA TCT GAA CTT C) [23], *CHK1 *(F: AGC GGT GGT CAA AAG AAT G, R: TGT CTG CAT CCA ATT TGG TAA), and *PPIA *(F: TTC ATC TGC ACT GCC AAG AC, R: TCG AGT TGT CCA CAG TCA GC).

### Combination index calculations

CompuSyn (ComboSyn, Inc., Paramus, NJ) was used to assess the interaction of the drugs in combination for synergy/additivity/antagonism to determine the combination index (CI) using the Chou-Talalay method [24]. Cell proliferation assay data (MTS assays) were expressed as the fraction of cells inhibited by the individual drugs or the combination compared to the vehicle (DMSO)-treated control cells; these data were used to determine CI [see Additional file 2, Table S2]. When the CI is <1, the combination is synergistic, when the CI is =1 the combination is additive, and when the CI is >1 the combination is considered antagonistic.

### Protein analysis

Cells were washed in PBS and lysed for protein in radio-immunoprecipitation assay (RIPA) buffer. Protein was quantified using a BCA protein assay kit (Pierce, Rockford, IL), separated by polyacrylamide gel electrophoresis and transferred to nitrocellulose membrane (Invitrogen) for detection using the following primary antibodies: RRM1 (3388; Cell Signal, Danvers, MA, USA), RRM2 (10846; Santa Cruz, Santa Cruz, CA, USA), β-tubublin (RB-9249; Thermo Scientific, Rockford, IL, USA ), Phospho-gamma H2AX (2577; Cell Signaling Technology, Beverly, MA), Cyclin A (Rb-1548; Neomarkers, Freemont, CA, USA) β-actin (A1978; Sigma), phospho Chk1 ser-345 (Rb-2348; Cell Signaling Technology), Chk1 (Ms-2360; Cell Signaling Technology).

### Cell cycle analysis

Actively growing MDA-MB-231 cells were pulsed with 10 μg/mL bromodeoxyuridine (BrdU) (BD Pharmingen, San Diego, CA, USA) for one hour. Cells were trypsinized, washed and fixed in 70% ethanol. DNA was denatured and cells were incubated with anti-BrdU-FITC (BD Pharmigen) for one hour. Cells were washed and re-suspended in 10 μg/mL propidium iodide (PI) solution (Sigma-Aldrich) to detect cell viability. Cells were sorted by flow cytometry, exciting at 488 nm and measuring BrdU-FITC with a 514 nm filter and PI with a 699 filter. Data points represent an average of at least three samples and the experiment was repeated twice.

### Apoptosis assay

Early and late apoptosis was detected in cells labeled with Annexin V- fluorescein isothiocyanate (FITC) and 7-amino-actinomycin D (7-AAD, eBioscience, San Diego, CA, USA). Briefly, cells were treated with the therapeutic agents indicated. At 48 hours, media was collected to retain floating cells and adherent cells were washed in PBS and trypsinized. Cell fractions were pooled, centrifuged, washed in PBS, and re-suspended in Annexin V binding buffer. Cells were incubated with Annexin V for 20 minutes, washed in PBS and re-suspended in Annexin V binding buffer. 7-AAD was added immediately prior to sorting by flow cytometry, excited at 488 nm with Annexin V-FITC levels measured with a 514 nm filter and 7-AAD with a 699 filter. Cells undergoing early apoptosis were detected by plasma membrane exclusion of viability dye 7-AAD and inclusion of Annexin-V. Late stage apoptosis was detected by plasma membrane inclusion of both 7-AAD and Annexin-V. Data points represent the average of four samples per treatment and the experiment was repeated twice.

### Caspase 3/7 Assay

Apoptosis was measured by Caspase 3/7 activation 48 hours after drug treatment. Caspase 3/7 substrate 100 μl (Caspase-Glo 3/7 Assay, Promega) was added to the cells for one hour and luminescence was measured by a Glomax luminometer using the standard Promega protocol.

### *In vivo *drug studies

Animal studies were approved and performed in accordance with the National Institutes of Health Intramural Animal Care and Use Program. Primary C3(1)/Tag mammary tumors or MDA-MB-231 xenograft tumors were isolated and prepared into 1 mm^3 ^fragments. Tissue fragments were implanted into the mammary fat pad (#4) of female SCID/NCr mice (BALB/c background) from the National Cancer Institute Animal Production Program (Frederick, MD, USA), n = 8/cohort. Drug treatment was initiated once tumors reached 125 mm^3^. Tumor size was monitored bi-weekly using caliper measurements (in millimeters) in two dimensions (length and width with tumor volume = (length × (width)^2^/2. For C3(1)Tag tumor bearing mice, gemcitabine (20 mg/kg) was delivered by intraperotineal (IP) injection on an every four days for three treatments (Q4Dx3) schedule. UCN-01 (4.5 mg/kg) was delivered by intravenous (IV) injection on a Q4x3 schedule with delivery scheduled twice per day, six hours apart (Q6Hx2) schedule. Mice treated with single or combination therapy received gemcitabine (or IP vehicle) first followed eight hours later by the first UCN-01 (or IV vehicle) treatment. For MDA-MB-231 tumor bearing mice, gemcitabine (5 mg/kg) and UCN-01 (6 mg/kg) were delivered as noted above except that UCN-01 (or IV vehicle) was first delivered 24 hours after gemcitabine (or IP vehicle) rather than eight hours later.

## Results

### Triple-negative breast cancer cells express the Tag signature

**Figure 1:**
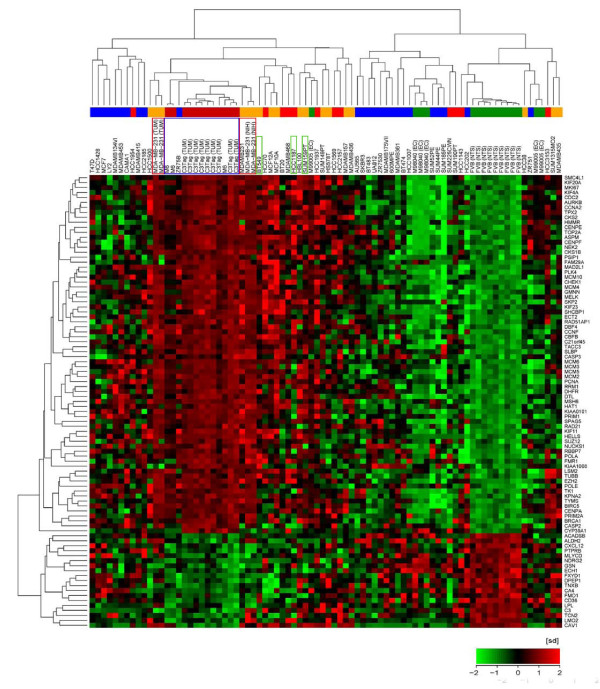
**Gene expression profile for the SV40/Tag signature across human breast cancer cell lines**. Triple negative MDA-MB-231 cells express a genetic profile for the SV40/Tag signature similar to C3(1)/Tag tumors. Two-way hierarchical clustering uses z-score transformed expression values within each microarray dataset, one minus un-centered correlation distance metric and complete linkage. The colored bar classifies the 51 cell lines from Neve *et al*. [16] and new samples into breast cancer subtypes adopted from Neve *et al*. [16]: Luminal (blue), Basal A (red), claudin-low (orange), and normal-like (green). MB-MDA-231 cells (red box) cluster closely with C3(1)/Tag tumors and M6 cell line (blue box). Other triple negative cell lines HCC 1187, SUM 159, and BT-549 are also highlighted (green boxes).

As previously reported, mammary tumors from C3(1)/Tag transgenic mice express a genetic signature that is highly represented in TNBCs [8]. In order to identify human breast cancer cell lines that are enriched for the Tag signature and that could be useful for determining the biological effects of knocking-down the expression of those genes in a preclinical model, gene expression data from the 51 breast cancer cell lines reported by Neve *et al*. [16] were analyzed for expression of the Tag signature. Additionally, gene expression profiles were also generated from RNA extracted from C3(1)/Tag mammary tumors, normal mammary tissue from FVB/N mice, M6 cells (a cell line derived from C3(1)/Tag mammary tumors) [12], and normal human breast epithelial cells M98040 and M99005 [13]. Mouse and human gene expression data were integrated and analyzed by hierarchical clustering using Z-score transformed expression values within each microarray dataset, the one minus un-centered correlation distance metric and complete linkage (GEO accession numbers: GSE25487 and GSE25488) (Figure 1). As predicted, C3(1)/Tag mammary tumors and M6 cells express the Tag signature whereas normal human breast epithelial cells and normal mammary tissue from FVB mice do not share expression of the Tag signature. Of the human breast cancer cell lines analyzed, the TNBC MDA-MB-231 cells most robustly expressed the Tag signature as evidenced by the tight clustering of these cells with Tag mammary tumors and the M6 cell line (Figure 1). For this reason, the MDA-MB-231 cell line was subsequently chosen as the human breast cancer model for comparative studies in this manuscript. The MDA-MB-231 cell line (MDA-MB-231 NIH) used in this study was also analyzed by microarray and shown to contain an expression signature quite similar to that as originally reported by Neve *et al*. [16].

### Identification of genes critical for proliferation of MDA-MB-231 cells using a siRNA-based screen

**Figure 2:**
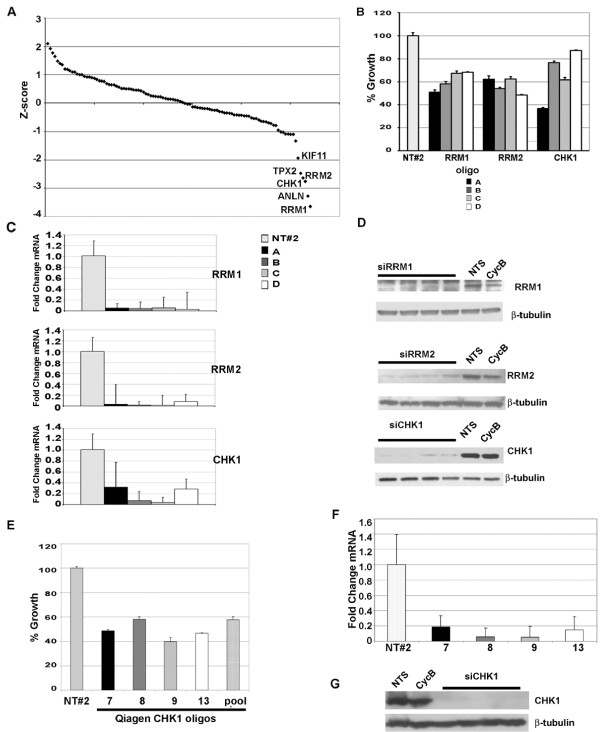
**siRNA screen for genes that promote proliferation of MDA-MB-231 cells identify CHK1, RRM1 and RRM2 as top-hits**. (**A**) Effects on proliferation by gene knockdown with the custom siRNA library. Data are shown as a z-score distribution from the mean. (**B**) Percent change (from non-targeting siRNA (NTS) control) in proliferation of cells due to knock-down of expression by individual siRNA oligos for the genes noted. Q-RT-PCR determination of reduced RNA (**C**) and protein (**D**) expression for CHK1, RRMI, and RRM2 for individual siRNA oligos. **(E) **Percent growth of cells due to expression of Qiagen CHK1-siRNA. Gene (**F**) and protein (**G**) expression for CHK1 is suppressed in cells that are transfected with individual Qiagen CHK1-siRNAs. Graphs shown are representative of 3 repeated experiments.

To identify genes within the Tag signature whose expression is critical for TNBC tumor survival and growth, a custom siRNA library was designed to target the 80 up-regulated genes contained in the Tag signature [see Additional file 1, Table S1]. Twenty-eight additional genes were also included in the siRNA library based upon genetic relevance to TNBC and elevated RNA expression in M6 cells and C3(1)/Tag tumors [see Additional file 1, Table S1]. MDA-MB-231 cells were reverse-transfected with the Tag siRNA library and assayed for changes in proliferation 72 hours later [see Additional file 1, Table S1; Figure 2A]. Out of 108 genes targeted by siRNA, the knockdown of six genes significantly inhibited cell growth as compared to cells transfected with a pool of non-targeting siRNA oligo controls (NTS) (Figure 2A). These six genes are *RRM1*, *RRM2*, *CHK1*, *TPX2*, Anillin (*ANLN*), and kinesin-related mitotic motor protein (*KIF11*) (Table 1). Two other human cell lines Hs578T, a TNBC cell line, and MCF10A, a non-tumorigenic mammary cell line considered to exhibit characteristics of normal mammary epithelial cells, were also screened using the custom siRNA library. The same six genes identified as critical to the growth of MDA-MB-231 cells also significantly inhibited proliferation of the TNBC cell line HS578T [see Additional file 1, Table S1; Table 1]. However, while siRNAs for *ANLN*, *TPX2 *and *KIF11 *significantly inhibited MCF10A cell proliferation, knock-down of *RRM1*, *RRM2 *and *CHK1 *did not [see Additional file 1, Table S1; Table 1]. These findings suggest that *RRM1*, *RRM2 *and *CHK1 *are selectively important for the TNBC cells, whereas *ANLN*, *TPX2*, and *KIF11 *may be important to cellular proliferation, but are not specific to triple negative breast tumorigenesis. In this study, we therefore focused on *RRM1*, *RRM2 *and *CHK1 *as targets for TNBC.

**Table 1:** Z-scores of top gene hits for siRNA screen in MDA-MB231 cells.

	MB231 (screen 1)	MB231 (screen 2)	Hs578T	MCF10A
ANLN	-3.275	-2.657	-2.139	-2.230
CHEK1	-2.766	-2.441	-4.719	-0.218
KIF11	-2.475	-2.231	-3.832	-2.770
RRM1	-2.637	-2.347	-2.053	-0.955
RRM2	-3.647	-2.915	-2.518	-1.348
TPX2	-1.932	-1.697	-3.864	-1.452

To confirm that the effect of pooled siRNA oligos was specific, the pooled siRNA oligos for *RRM1*, *RRM2*, and *CHK1 *genes were de-convoluted and each of the four individual siRNAs was reverse-transfected into MDA-MB-231 cells and assayed for inhibition of proliferation (Figure 2B). In comparison to a non-targeting control siRNA (NT#2) at 100% growth, the four siRNA oligos for *RRM1 *and *RRM2 *individually resulted in a 30% to 50% inhibition of proliferation. Those for *CHK1 *individually inhibited proliferation by 12% to 60%. The individual siRNA oligos for *RRM1*, *RRM2 *and *CHK1 *produced a marked reduction in gene (Figure 2C) and protein expression (Figure 2D). To further confirm that *CHK1 *was not selected through off target effects, MDA-MB-231 cells were transfected with pooled siRNAs from another commercial source (Qiagen) and assayed for their effects on proliferation (Figure 2E). Cell proliferation was significantly inhibited by siRNA silencing of *CHK1 *using these oligos. De-convolution of this second siRNA pool demonstrated that all four of these siRNA oligos significantly inhibited proliferation by at least 40% and reduced *CHK1 *RNA (Figure 2F) and protein (Figure 2G) expression.

### Combination therapy with UCN-01 and gemcitabine induces DNA damage and apoptosis in triple negative breast cancer cell lines

Identification of *RRM1*, *RRM2*, and *CHK1 *in the siRNA screen as critical genes for proliferation of MDA-MB-231 cells suggests that these genes may be effective therapeutic targets for TNBC. Because RRM1 and RRM2 comprise the two subunits of ribonucleotide reductase, an enzyme essential for maintaining adequate levels of dCTPS for DNA synthesis and DNA repair, and CHK1 inhibits cell cycle progression to allow for adequate DNA damage repair [25], we hypothesized that the combined inhibition of all three proteins together could synergize and enhance the inhibitory effects on cell proliferation observed with each target individually. To address this question, we treated the human MDA-MB-231 and mouse M6 (derived from a C3(1)/Tag mammary tumor) TNBC cell lines either alone or in combination with gemcitabine, a drug that inhibits ribonucleotide reductase activity and subsequently promotes DNA damage [26], and with UCN-01, a CHK1 inhibitor [27] that prevents cell cycle arrest leading to deficient DNA repair.

To determine whether gemcitabine is an effective inhibitor of TNBC cell proliferation as a single agent, MDA-MB-231 and M6 cells were tested in triplicate with at least eight different doses over a concentration range of 10 μM to 1 μM and assayed for changes in proliferation. In keeping with a previous report [28], MDA-MB-231 cells were sensitive to gemcitabine with an average IC_50 _of 20 nM. M6 cells were also sensitive to gemcitabine with an average IC_50 _of 3.9 nM. To determine whether UCN-01 is an effective inhibitor of TNBC cell proliferation as a single agent, MDA-MB-231 and M6 cells were treated with UCN-01 with at least eight doses over a concentration range of 50 ρM to 1 μM and assayed for inhibition of proliferation. MDA-MB-231 and M6 cells treated with UCN-01 have average IC_50 _values of 173 nM and 22 nM, respectively.

**Figure 3:**
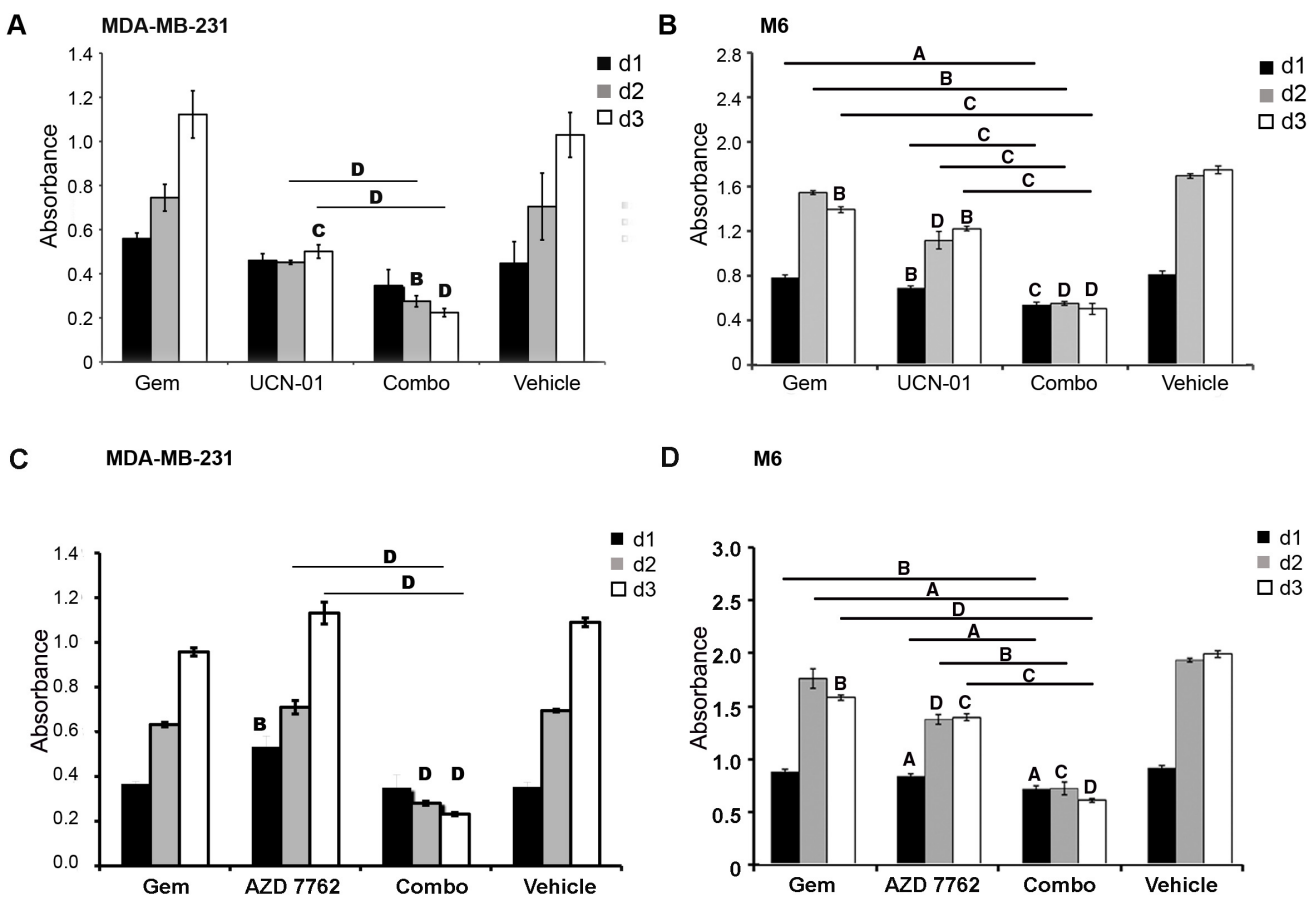
**Combination therapy with CHK1 inhibitors and gemcitabine inhibits proliferation in TNBC cells**. MDA-MB-231 (**A, C**) and M6 (**B, D**) cells were treated with agents on day 0 and proliferation was measured on days indicated. MDA-MB-231 cells (gemcitabine 10 nM; UCN-01 150 nM), (gemcitabine10 nM; AZD 7762 200 nM). M6 cells (gemcitabine 4 nM; UCN-01 20 nM) (gemcitabine 4 nM; AZD 7762 30 nM). Results in B and D are from one experiment thus Gem treatment and vehicle are the same in both panels. *P*-value based upon change from vehicle treatment (letters only) and from single agent to combination treatment (line and letter) (A ≤ 0.01, B ≤ 0.005, C ≤ 0.001, D ≤ 0.0005).

To determine whether combination treatment of gemcitabine and UCN-01 resulted in a greater inhibition of proliferation than either single agent, MDA-MB-231 (Figure 3A) and M6 cells (Figure 3B) were treated with gemcitabine and UCN-01 either alone or in combination at sub IC_50 _concentrations and proliferation was measured over three days. Compared to MDA-MB-231 cells treated with vehicle, treatment with 10 nM gemcitabine alone did not significantly reduce cell proliferation over three days. Cells treated with 150 nM UCN-01 significantly reduced proliferation by day three. However, MDA-MB-231 cells treated with both drugs in combination showed the greatest inhibition of proliferation in comparison to vehicle-treated cells on days two and three. Since UCN-01 exerts a greater inhibitory effect than gemcitabine treatment at these concentrations, a comparison between UCN-01 and the combination treatment determined that the dual treatment inhibits growth more than UCN-01 alone on days two and three. M6 cells were also significantly growth inhibited by UCN-01, gemcitabine (*P *= 0.02 for days one and three), and by combination dosing with gemcitabine and UCN-01.

### Gemcitabine and UCN-01 inhibit TNBC cell growth synergistically

To determine whether the interaction of gemcitabine and UCN-01 was synergistic or merely additive, the interactions were evaluated by the Chou-Talalay CI method [24]. In this method, a CI <1 represents synergism, whereas a CI = 1 represents an additive effect and a CI >1 represents antagonism. The CIs in all combination treatments were less than 1 [see Additional file 2, Table S2A], confirming that treatment with gemcitabine and UCN-01 had a synergistic cytotoxic effect. Likewise, with the M6 cells the combination treatment inhibited proliferation more potently than either drug alone (CI <1, Additional file 2, Table S2B).

To confirm that the effects of UCN-01 are related to CHK1 inactivation, cells were also dosed alone and in combination with sub IC_50 _concentrations of gemcitabine and AZD 7762, an ATP-competitive checkpoint kinase inhibitor [11] , and assayed for proliferation (Figure 3C and 3D). Similarly, MDA-MB-231 (IC_50 _for AZD 7762 = 120 nM) and M6 (IC_50 _for AZD 7762 = 36 nM) cells were growth inhibited when treated with gemcitabine and this CHK1 inhibitor, suggesting that subIC_50 _dosing of drugs that target RRM1/RRM2 and CHK1 could be an effective therapy regimen for TNBC.

It has been established that gemcitabine exerts anti-tumor activity through two different mechanisms. Gemcitabine inhibits RRM1 and RRM2 leading to inhibition of nucleoside synthesis and DNA replication; it can also be directly incorporated into replicating DNA leading to the termination of DNA strand synthesis. To test whether CHK1 inhibition worked synergistically with another chemotherapeutic agent that damages DNA through cross-linking, we performed similar experiments using a combination of UCN-01 and cisplatin. Interestingly, we did not observe a synergistic effect using this drug combination with MDA-MB-231 cells (data not shown).

### Combination therapy increases DNA damage associated with inhibition of cell cycle progression

**Figure 4:**
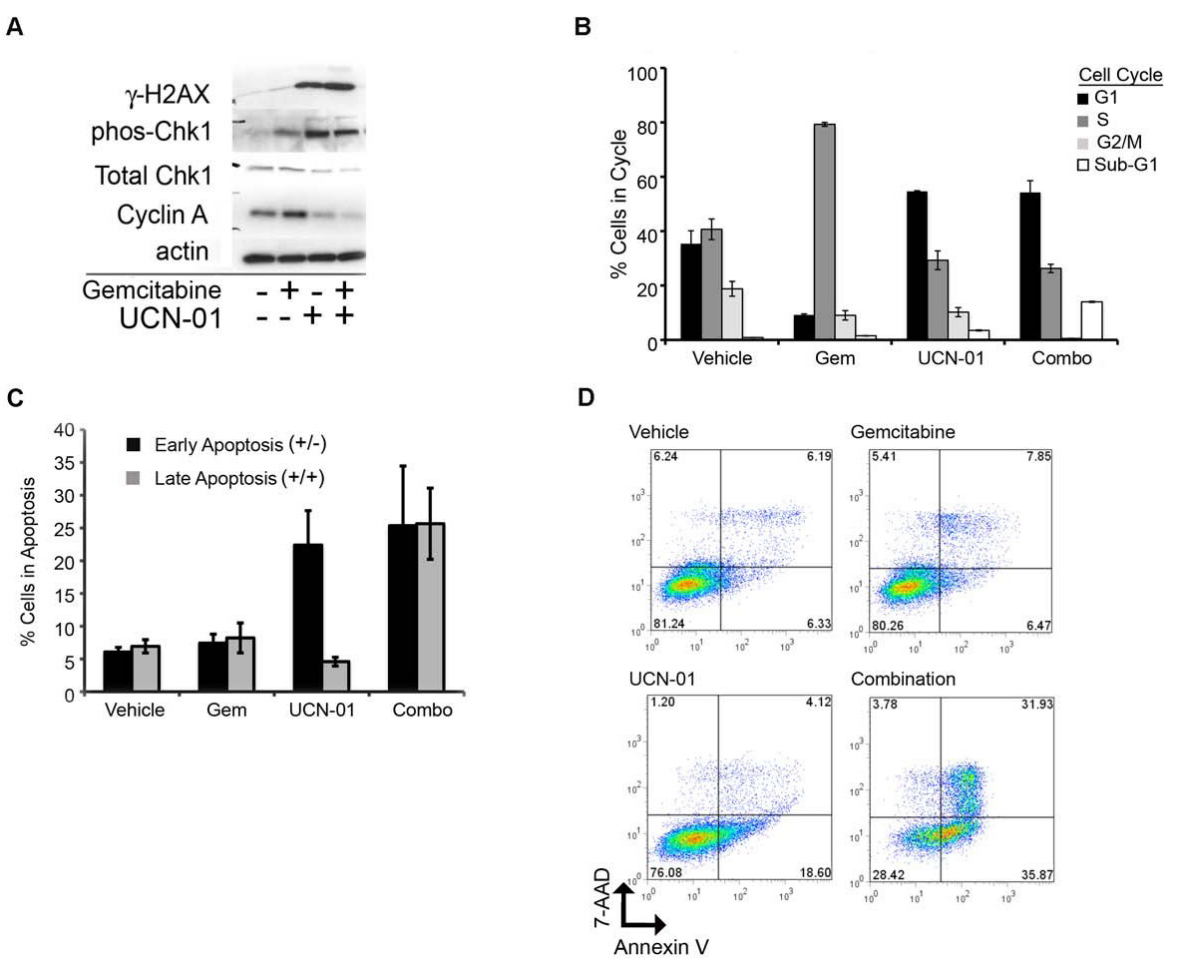
**Combination therapy with UCN-01 and gemcitabine induces DNA damage and apoptosis in TNBC cells**. (**A**) Protein samples were collected 24 hours after drug treatment to detect changes in DNA damage (gamma-H2AX), checkpoint activation (phos-CHK1 andtTotal CHK1) and cell cycle progression (Cyclin A) by immunoblot analysis. (**B**) Cell cycle changes were assessed by BrdU-labeling and propidium iodide staining 24 hour after drug treatment. (**C**) Percentage of cells at 48 hours in early apoptosis (Annexin V ^+^/7-AAD^-^) and late apoptosis (Annexin V+/7-AAD+) with representative data (**D**). MDA-MB-231 cells (gemcitabine 10 nM; UCN-01 150 nM), M6 cells (gemcitabine 4 nM; UCN-01 20 nM). *P*-value based upon change from vehicle treatment (A ≤ 0.01, B ≤ 0.005, C ≤ 0.001, D ≤ 0.0005).

To interrogate how the combination treatment enhances cell death, MDA-MB-231 cells were treated with gemcitabine and UCN-01 individually, and in combination, and harvested for protein 24 hours later (Figure 4A). Immunoblot analyses revealed that DNA damage, as determined by phos-gamma-H2AX and phos-CHK1 expression, was minimally induced by gemcitabine. However, treatment with UCN-01 as a single agent resulted in increased DNA damage, which was further increased when UCN-01 was given in combination with gemcitabine. Total CHK1 protein expression was reduced with UCN-01 and combination treatment at 24 hours. As Cyclin A protein expression is highest in S-phase and decreases with progression through the cycle, cell cycle progression was stalled in S-phase by gemcitabine, as demonstrated by the accumulation of Cyclin A protein compared to control treated cells. However, cells treated with UCN-01 alone or in combination with gemcitabine exhibited reduced Cyclin A protein expression compared to control-treated cells indicating cell cycle progression through G2/M. BrdU incorporation into proliferating MDA-MB-231 cells 24 hours after drug treatment revealed that cell cycle progression was disrupted by all drug treatments. In 79% of the cells treated with gemcitabine alone there was a dramatic increase in the S phase component of the cell cycle (79%) compared to vehicle treated cells (40%) at 24 hours. UCN-01 promotes cell cycle progression at the G2/M checkpoint resulting in an approximately two-fold decrease in the G2/M component and a 50% increase in the G1 fraction (54%) compared to vehicle treated cells (37%). Fifty-four percent of the cells treated with both drugs were in the G1 fraction and a significant, approximately three-fold increase in the sub-G1 fraction of cells (14%) was observed compared to UCN-01 alone (4%) (gemcitibine - 2%; vehicle - 1%) suggesting that a large portion of combination treated cells were undergoing cell death (Figure 4B).

To determine whether the combination therapy increased apoptosis, MDA-MB-231 cells were treated with the drugs and assayed for apoptosis by Annexin V-FITC/7-AAD staining at 48 hours. Annexin V-FITC positive/7-AAD negative (early apoptosis) and Annexin V positive/7-AAD positive (late apoptosis) labeling further supported the finding that combination dosing of gemcitabine and UCN-01 enhanced cell death at subIC_50 _concentrations of the individual drugs (Figures 4C and 4D).

### Other triple negative cell lines respond to the combination treatment

**Figure 5:**
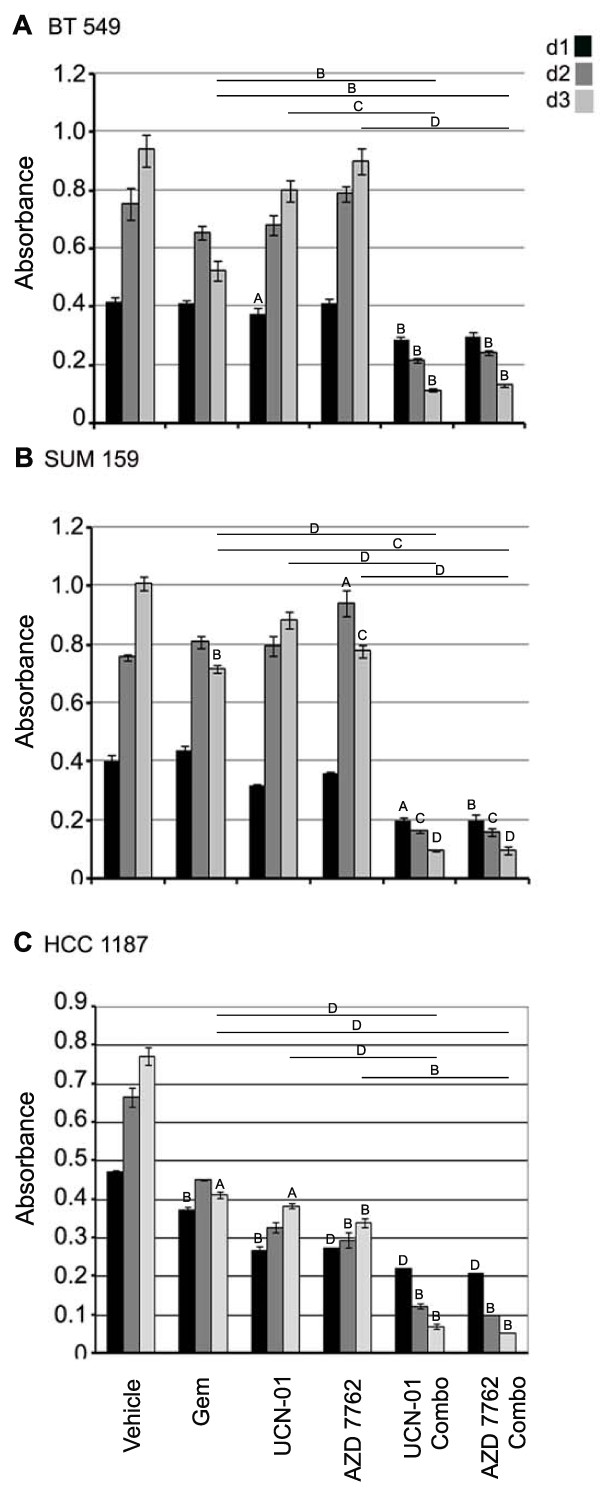
**Combination therapy with CHK1 inhibitors and gemcitabine inhibits proliferation in TNBC cells**. BT-549 (**A**), SUM 159 (**B) **and HCC 1187 **(C**) cells were treated with agents on day 0 and proliferation was measured on days indicated. BT-549 cells (gemcitabine 10 nM; UCN-01 100 nM; AZD 7762 150 nM). SUM 159 (gemcitabine 4 nM; UCN-01 80 nM; AZD 7762 300 nM). HCC 1187 (gemcitabine 10 nM; UCN-01 150 nM; AZD 7762 200 nM). *P*-value based upon change from vehicle treatment (letters only) and from single agent to combination treatment (line and letter; day three only) (A ≤ 0.01, B ≤ 0.005, C ≤ 0.001, D ≤ 0.0005).

The response of the MDA-MB-231 cell line to the gemcitabine and CHK1 inhibitor combination therapy supports the idea that RRM1 and 2 and CHK1 are good targets for triple negative tumors that overexpress these genes. To determine whether other triple negative cell lines respond to the gemcitabine-CHK inhibitor combination treatment, cell lines BT-549, HCC 1187, and SUM 159 were tested. As described previously, cells were first treated with gemcitabine, UCN-01 or AZD 7762 over a dose range to determine the IC_50 _concentrations of the single agents (Table 2). The three cells lines were then treated with sub IC_50 _concentrations alone and in combination with the agents as noted previously. Proliferation of all three cell lines was significantly inhibited by the combination treatments (Figure 5A-C). The combination treatments were synergistic [see Additional file 2, Tables S2C-E]. Moreover, the combination treatment increased the level of apoptosis as measured on day two by a Caspase 3/7 assay [see Additional file 3, Figure S1A-C].

**Table 2:** IC_50 _Concentrations for other human triple negative breast cancer cell lines.

	BT- 549	SUM 159	HCC 1187
Gemcitabine	13.5 nM	5.27 nM	17.1 nM
UCN-01	160.8 nM	116.4 nM	230.5 nM
AZD 7762	275.2 nM	509.1 nM	307.7 nM

In addition, to determine whether the non-tumorigenic human MCF-10A cell line and the ER+ MCF-7 tumorigenic cell line also responded to this combination therapy, IC50 concentrations were determined and cells were treated with <IC50 concentrations of the drugs as described above. Combination therapy did inhibit the proliferation of both of these cell lines more than that observed for each drug alone [see Additional file 4, Figure S2. This is not necessarily surprising given the mechanism of action of these agents and the fact that these cell lines have been selected to replicate relatively rapidly under tissue culture conditions. Inhibition of the cell cycle checkpoint with the induction of DNA damage in rapidly dividing cells would lead to inhibition of cell growth and apoptosis. However, unlike the response of the TNBC cells to the combination therapy where there was overwhelming cell death, the combination therapy treatment of MCF10A and MCF7 cells resulted in an initial reduction in cell numbers, but a gradual recovery of cell growth over three days.

### Expression of CHK1, RRM1 and RRM2 is not correlated with p53 or Rb status

Since the gene signature we used in this study is in large part related to the loss of p53 and Rb functions which are typically lost in TNBC, we determined whether there was a correlation between loss of p53 or Rb function and expression of CHK1 or RRM1/2 in the microarray data of the panel of 51 cell lines (including MCF10A and MCF12A) reported by Neve *et al*. according to their known p53 status. Cell lines were separated into two groups dependent upon whether they harbor wild-type or mutated/deleted p53. We found no statistical difference in *RRM1 *or *CHEK1 *gene expression in cells with wild type or mutated p53 protein (unpaired two-tailed t-test, *P *= 0.456 for *CHEK1 *and 0.887 for *RRM1*). Pearson's correlation analysis of *RRM1 *or *RRM2 *or *CHEK1 *gene expression was performed in association with Rb gene expression or its regulator CDKN21 (p16 CDK inhibitor). We also did not find a correlation between expression of Rb or p16 and any these genes. The correlation coefficients were ranging from 8E-5 to 0.057.

### Combination therapy of gemcitabine with UCN-01 inhibits growth of TNBC tumor xenografts

To determine whether gemcitabine and UCN-01 inhibits growth of triple negative breast tumors *in vivo*, we utilized two mouse models, the C3(1)/Tag mouse mammary tumor transplant model (previously established in our lab) and the MDA-MB-231 human tumor xenograft model. Freshly isolated 1 mm^3 ^tumor fragments were excised from C3(1)/Tag transgenic mice or MDA-MB-231 tumor xenograft bearing mice and transplanted into the mammary glands of SCID mice. Once tumors grew to approximately 125 mm^3 ^about eight to ten days later, mice were dosed with gemcitabine by IP injection on a Q4Dx3 schedule or UCN-01 by IV injection on a Q8Hx2 schedule repeated every other day for six days. Initial screening to establish dose ranges revealed that both models are sensitive to gemcitabine with tumor inhibition at doses greater than 40 mg/kg. However, both models had poor tumor sensitivity to UCN-01 at doses of 4.5 and 6.67 mg/kg (data not shown).

**Figure 6:**
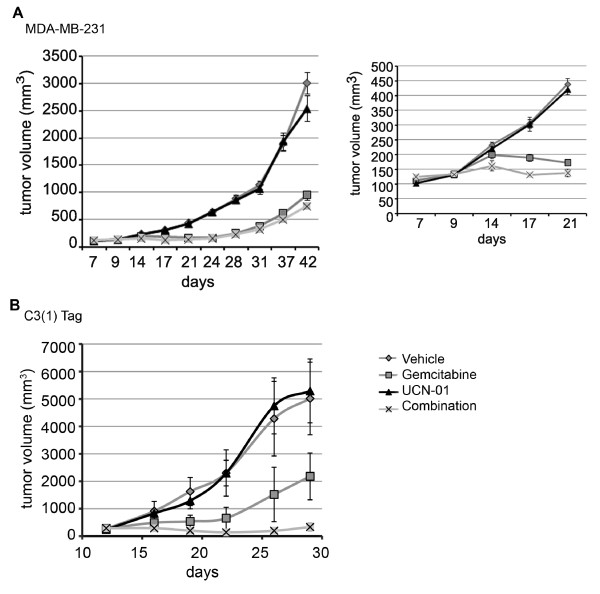
***In vivo *response of TNBC tumors to agents that target CHK1, RRM1 and RRM2**. (**A**) SCID mice with MDA-MB-231 tumor xenografts were treated seven days after implantation with vehicle, 5 mg/kg gemcitabine, 6 mg/kg UCN-01, or a combination of both on a Q4Dx3 schedule. Gemcitabine was delivered first by IP injection and UCN-01 was delivered 24 hours later by IV injection on a Q6Hx2. Note: Inset graph highlights days 7 to 21. During this period, mice treated with the combination therapy had slower growing tumors than those treated only with gemcitabine. (**B**) SCID mice with C3(1)Tag tumor transplants were dosed 12 days after transplantation with 20 mg/kg gemcitabine, 4.5 mg/kg UCN-01, or the drug combination on a Q4Dx3 schedule. Gemcitabine was delivered first and UCN-01 was delivered eight hours later on a Q6Hx2 schedule.

To determine whether combination treatment of UCN-01 and gemcitabine reduced tumor growth better than gemcitabine alone, SCID mice with MDA-MB-231 tumor xenografts were treated seven days after tumor implantation with vehicle, 5 mg/kg gemcitabine, 6 mg/kg UCN-01, or a combination of both on a Q4Dx3 schedule. Gemcitabine was delivered first by IP injection to induce DNA damage and UCN-01 was delivered 24 hours later by IV injection on a Q6Hx2 schedule to block DNA damage repair. MDA-MB-231 tumor growth, measured twice weekly, was not affected by treatment with vehicle or UCN-01 as a single agent, but it was significantly inhibited by treatment with gemcitabine alone (Figure 6A). Combination treatment initially resulted in a modest further inhibition of tumor growth compared to gemcitabine treatment alone, which was not sustained once therapy was discontinued. (Figure 6A inset)

SCID mice with C3(1)/Tag tumor fragments were dosed 12 days after transplantation with 20 mg/kg gemcitabine, 4.5 mg/kg UCN-01, or the drug combination on a Q4Dx3 schedule. Gemcitabine was delivered first and UCN-01 was delivered eight hours later on a Q6Hx2 schedule. Mice treated with vehicle showed exponential growth over eight days (Figure 6B). Treatment with UCN-01 did not inhibit tumor growth while gemcitabine significantly reduced tumor growth. Mice treated with both UCN-01 and gemcitabine showed remarkable inhibition in tumor growth up to the end of the study period at day 30 compared to the single agent treatments.

## Discussion

This study has explored the functional significance of genes within a previously described Tag expression signature, which includes many genes dysregulated through the loss of p53 and Rb function and which is highly expressed in human TNBC [8]. Based upon an analysis of gene expression of 51 human breast cancer cell lines, we identified MDA-MB-231 cells as a TNBC cell line that robustly expresses the Tag signature. We designed a human siRNA library to knock-down the up-regulated genes in the Tag signature and utilized the high-throughput readout of proliferation changes to efficiently screen for genes whose loss of function significantly reduced cell growth. The identification of RRM1, RRM2 and CHK1 as key regulators of TNBC growth suggests that this screening method is an effective tool for identifying potential drugable targets. Our further validation of these candidates in other cell lines and xenograft models suggests that we have identified a potentially useful drug combination, gemcitabine and a CHK1 inhibitor, for treatment of TNBC. While some BrCa patients are treated with gemcitabine, the addition of a CHK1 inhibitor may offer the possibility of achieving a better therapeutic response or an improved response using a lower dose of gemcitabine for patients who may be more susceptible to the side effects of gemcitabine.

A major concern for novel drug therapies is acquired drug resistance and it is thought that combination therapies are more effective than single agents [29]. We utilized the availability of small molecule inhibitors of CHK1 (UCN-01 and AZD 7762) and RRM1 and RRM2 (gemcitabine), to test the efficacy of these compounds on human TNBC cells as well as the M6 cell line derived from the C3(1)/Tag mammary tumors. By inhibiting both CHK1 and ribonucleotide reductase, we saw a superior inhibition of proliferation *in vitro *and tumor growth *in vivo*. We hypothesized that the inhibition of CHK1 in the context of inducing DNA damage through the disruption of RRM1 and RRM2 function by gemcitabine could significantly augment the killing capacity of gemcitabine. Our *in vitro *results indicated that this was the case. The combination treatment of UCN-01 and gemcitabine led to both a reduction of CyclinA - indicating a loss of stalling in S-phase and movement through the G2/M checkpoint and increased DNA damage as reported by an increase in phospho-gamma-H2AX expression. Fluorescence activated cell sorting (FACS) analysis further revealed that the combination therapy drove cells through the G2/M checkpoint and induced significant apoptosis. We also demonstrated that by combining UCN-01 with gemcitabine, a lower dose of gemcitabine could be used to kill the tumor cells, which has translatable potential to the clinic. Our *in vivo *results demonstrated that inhibition of CHK1 alone did not have a striking effect on tumor development, although gemcitabine was quite growth inhibitory to the tumors. In fact, MDA-MB-231 tumor growth was almost completely abrogated by gemcitabine alone. During the course of actual drug treatment, the combination inhibited tumor growth better than gemcitabine alone. However, once treatment was discontinued, the effect was not as obvious. This suggests that alternative dosing schedules may improve the efficacy of this combination therapy.

The value of genetically-engineered mouse models of human cancer for preclinical studies has been demonstrated for particular cancer models and drugs [30]. Based upon this study, the C3(1)/Tag model exhibits important molecular similarities to human TNBC that can be used to test targeted therapies. An important advantage of using this transgenic model is that studies can be performed in animals with an intact immune system unlike model systems that use human xenografts. Given the importance of the immune system in regulating tumor development, this model system will allow for novel combination therapies that may inhibit particular targets critical to the growth and survival of TNBC along with anti-cancer therapies that alter the immune response. We have recently developed a novel, syngeneic transplantable model system for C3(1)/Tag tumors that will allow for the more rapid screening of therapies using this model of TNBC (manuscript in preparation).

Our findings correlate well with a recent report that identified CHK1 as being over-expressed in TNBCs and that p53 expression does not directly effect CHK1 expression [31] suggesting that CHK1 is a valid TNBC target, distinct from other non-tumor cells. Our analyses did not find a correlation between loss of p53 or Rb function and increased expression of CHK1 or RRM1/2 suggesting that the increased expression is not a direct result of the loss of these tumor suppressor activities. Although we used UCN-01 as a CHK1 inhibitor for most of our studies, it is known that UCN-01 binds to human plasma proteins [32,33] and thus has not been successful in the clinic. There are several other CHK1 inhibitors in development that are being tested in clinical trials [25] to determine whether they have better bio-availability and target specificity. One such agent is AZD 7762, which we used to confirm our *in vitro *results but did not have ample supply available for animal studies. Gemcitabine and CHK1 inhibitor (AZD 7762) are currently being tested in clinical trials as a combination therapy for late stage cancers. The results of our study suggest that this combination may be quite efficacious for patients with TNBC, or other patients whose tumors overexpress CHK1, RRM1 and RRM2. A Phase I clinical trial using the combination of UCN-01 and a topoisomerase inhibitor that also induces DNA damage has recently been reported for resistant solid tumor malignancies with suggestion of a positive response in two patients with TNBC [34].

It is possible that this combination therapy may be of value in other subtypes of BrCa which will need to be elucidated in future studies and where predictive biomarkers would identify patients who may respond to this therapy. It is also possible that baseline levels of CHK1 expression may not be the sole determining factor for efficacy of a CHK1 inhibitor, but rather the response of CHK1 expression in a tumor to a chemotherapeutic agent may be an important factor in defining the usefulness of a CHK1 inhibitor. Thus, tumors with low baseline levels of CHK1 may still benefit from a CHK1 inhibitor if CHK1 becomes elevated in response to a chemotherapeutic agent. This possibility should be explored in future studies.

## Conclusions

In summary, the functional analysis of genes contained within an expression signature originally identified through genomic cross-species analysis identified CHK1, RRM1 and RRM2 as potential targets for therapy. Combination therapy that inhibits both of these pathways showed a strong synergistic effect and may have translational value in treating human TNBC patients. Importantly, using relevant models of TNBC, we demonstrate *in vivo *that this combination therapy does result in a greater anti-tumor effect than either agent alone. The results of this study demonstrate that a 'subtype specific' gene expression signature, first identified through genomic analyses of genetically-engineered mouse (GEM) models of human cancer, can be valuable to rationally screen for drug targets and combination therapies. The validation of therapies in a standard xenograft model and a highly relevant GEM model of TNBC provides further support for considering this combination therapy in human clinical trials.

## Abbreviations

ANLN: anillin; BrdU: bromodeoxyuridine; C3(1): rat prostatein promoter; CHK1: checkpoint kinase 1; CI: combination index; Dhfr: dihydrofolate reductase; (D)MEM: (Dulbecco's) modified Eagle's medium; ER: estrogen receptor; FBS: fetal bovine serum; GEM: genetically-engineered mouse; HER2/ERRB2: v-erb-b2 erythroblastic leukemia viral oncogene homolog 2; Kif1: kinesin KIF1; Mcm: mini-chromosome maintenance; PBS: phosphate-buffered saline; pCR: pathologic complete response; PCR: polymerase chain reaction; Pk2: protein kinase 2; PR: progesterone receptor; pRb: retinoblastoma gene; p53: protein 53 tumor suppressor gene; Pola1: polymerase (DNA directed): alpha 1: catalytic subunit; RIPA: radioimmunoprecipitation assay; Rrm1: ribonucleotide reductase 1; Rrm2: ribonucleotide reductase 2; RT: room temperature; RT-PCR: reverse transcriptase-polymerase chain reaction; siRNA: small interfering RNA; Stmn1 stathmin 1; Tag: simian virus40-early region; TNBC: triple negative breast cancer; Top2a: topoisomerase 2a; TPX2: microtubule-associated: homolog (Xenopus laevis); Tyms: thymidylate synthetase.

## Competing interests

The authors declare that they have no competing interests.

## Authors' contributions

CNB designed the drug and *in vivo *studies, performed experiments, analyzed data, interpreted results, prepared figures, drafted the manuscript, and edited and revised manuscript. CCT designed the siRNA and drug studies, performed experiments, analyzed data, interpreted results, prepared figures, drafted the manuscript, and edited and revised manuscript. AMM analyzed microarray data, interpreted results and prepared figures. IMC designed and performed cell cycle experiments, analyzed data and interpreted results. DL designed and performed apoptosis experiments, analyzed data and interpreted results. LRM designed and performed drug studies and analyzed data. OA designed and performed drug studies and analyzed data. JS performed drug studies and analyzed data. HPW designed drug studies, and edited and revised the manuscript. NJC designed the siRNA studies, and edited and revised the manuscript. MGH designed *in vivo *drug studies, performed experiments, analyzed data, interpreted results and prepared figures. JEG designed research, interpreted results, drafted the manuscript, and edited and revised the manuscript. All authors read and approved the final manuscript.

## Supplementary Material

Additional file 1 **Table S1**. Custom siRNA oligo pools used for screening tumor cells. Click here for file

Additional file 2 **Table S2**. Combination Index data for drug synergy in triple negative cells. Click here for file

Additional file 3 **Figure S1**. Combination therapy with Chk1 inhibitors and gemcitabine increases apoptosis in TNBC cells. BT-549 (**A**), SUM 159 (**B) **and HCC 1187 **(C**) cells were treated with agents on day 0 and apoptosis was measured by Caspase 3/7 assay on day two. BT-549 cells (gemcitabine 10 nM; UCN-01 100 nM; AZD 7762 150 nM). SUM 159 (gemcitabine 4 nM; UCN-01 80 nM; AZD 7762 300 nM). HCC 1187 (gemcitabine 10 nM; UCN-01 150 nM; AZD 7762 200 nM). *P*-value based upon change from vehicle treatment (letters only) and from single agent to combination treatment (line and letter) (A ≤ 0.01, B ≤ 0.005, C ≤ 0.001, D ≤ 0.0005) Click here for file

Additional file 4 **Figure S2**. Combination therapy in normal and ER+ breast cancer cells reduces proliferation. The non-tumorigenic mammary cell line MCF10A **(A) **and the ER+ breast cancer cell line MCF-7 **(B) **were treated with individual agents or combination on day 0 and proliferation was measured by MTS assay on days indicated. MCF10A cells (gemcitabine 4 nM; UCN-01 20 nM), (gemcitabine 4 nM; AZD 7762 300 nM). MCF-7 cells (gemcitabine 8 nM; UCN-01 25 nM), (gemcitabine 8 nM; AZD 7762 50 nM). *P*-value based on change from vehicle treatment for all days (letters only) and from single agent to combination treatment, day three only (line with letter) (A ≤ 0.01, B ≤ 0.005, C ≤ 0.001, D ≤ 0.0005). Click here for file
