# Supplementary material for: Cross-species genomic and functional analyses identify a combination therapy using a CHK1 inhibitor and a ribonucleotide reductase inhibitor to treat triple-negative breast cancer
Source: Breast Cancer Res. 2012 Jul 19;14(4):R109. doi: 10.1186/bcr3230 (PMC3680937; doi:10.1186/bcr3230)
Supplement: Additional file 1 — Table S1. Custom siRNA oligo pools used for screening tumor cells. [file bcr3230-S1.PDF]

Supplementary Table 1 Custom siRNA oligo pools

Gene Symbol	Description	Accession #	Z-score MB231	Z-score Hs578T	Z-score MCF10A
AKT3	*thymoma viral proto-oncogene 3*	NM_005465	-0.184	-0.402	1.400
ANLN	*Anillin, actin binding protein*	NM_018685	-3.275	-2.139	-2.230
AREG	*amphiregulin*	NM_001657	1.197	-0.482	1.217
ASK	*Activator of S phase kinase*	NM_006716	0.519	-1.210	-0.515
AURKB	*Aurora kinase B*	NM_004217	-0.641	-0.572	0.191
BID	*BH3 interacting domain death agonist*	NM_197967	0.242	0.046	0.958
BIRC5	*Baculoviral IAP repeat-containing 5, transcript variant 3*	NM_001012270	-0.719	-1.489	-0.732
BMI1	*Bmi1 polycomb ring finger oncogene*	NM_005180	0.459	-0.441	0.045
BRCA1	*Breast cancer 1*	NM_007298	0.022	0.751	0.515
BUB1	*Budding uninhibited bybenzimidazoles 1 homologue*	NM_004336	0.776	-1.176	-0.471
C14ORF114	*Shugoshin-like 1*	NM_018199	1.770	-1.223	0.810
CASP3	*Caspase 3*	NM_032991	2.107	-1.414	-0.058
CBFB	*Core binding factor beta,*	NM_001755	1.024	-0.596	-0.054
CCNA2	*Cyclin A2*	NM_001237	-0.125	1.223	0.534
CCNE1	*Cyclin E1*	NM_001238	0.251	-0.957	-0.410
CCNF	*Cyclin F*	NM_001761	0.920	-0.879	0.474
CDC2	*Cell division cycle 2 homolog A*	NM_001786	-0.338	-0.462	0.139
CDCA3	*Cell division cycle associated 3*	NM_031299	-0.542	0.724	0.732
CDKN1B	*cyclin-dependent kinase inhibitor 1B*	NM_004064	1.487	-0.980	0.556
CENPA	*Centromere autoantigen A*	NM_001042426	0.480	-1.421	-0.958
CENPE	*Centromere associated protein-E*	NM_001813	-0.242	-3.278	-1.101
CENPF	*centromere protein F*	NM_016343	0.698	-0.145	0.580
CHEK1	*Checkpoint kinase 1 homolog*	NM_001274	-2.766	-4.719	-0.217
CKS1B	*CDC28 protein kinase 1b*	NM_001826	-0.130	-1.382	-0.767
CKS2	*CDC28 protein kinase regulatory subunit 2*	NM_001827	0.946	-1.005	-0.381
CXADR	*Coxsackievirus and adenovirus receptor*	NM_001338	1.410	-0.139	0.737
CYP39A1	*Cytochrome P450, family 39, subfamily a, polypeptide 1*	NM_016593	0.492	-1.062	-0.191
DHFR	*Dihydrofolate reductase*	NM_000791	-1.080	-0.148	-0.714
ECT2	*Ect2 oncogene,*	NM_018098	0.571	-0.296	0.984
EGFR	*epiderman growth factor receptor*	NM_201282	0.510	-0.952	-0.186
EGR1	*early growth response 1*	NM_001964	-0.192	-1.162	0.685
EZH2	*Enhancer of zeste homolog 2*	NM_152998	0.924	-1.335	-0.902
FAM29A	*Homo sapiens family with sequence similarity 29*	NM_017645	-0.040	-0.290	-1.470
FMR1	*Truncated fragile X mental retardation protein*	NM_002024	0.304	-1.290	-1.262
FOXM1	*Forkhead box M1*	NM_021953	-1.019	-0.625	-0.760
FZD2	*frizzled homolog 2*	NM_001466	-0.791	-1.585	-1.680
GMNN	*Geminin*	NM_015895	0.873	-0.609	1.290
H2AFZ	*H2A histone family, member Z*	NM_002106	-0.463	0.730	0.160
HAT1	*Histone aminotransferase 1*	NM_001033085	0.770	0.251	0.431
HELLS	*Helicase, lymphoid specific*	NM_018063	0.898	-1.045	0.337
HES1	*hairy and enhancer of split 1*	NM_005524	1.361	-0.880	1.177
HEY1	*hairy/enhancer-of-split related with YRPW motif 1*	NM_012258	1.205	-0.041	2.111
HMMR	*Hyaluronan mediated motility receptor (RHAMM)*	NM_012484	0.525	-0.593	0.362

Gene Symbol	Description	Accession #	Z-score MB231	Z-score Hs578T	Z-score MCF10A
IL13RA1	*interleukin 13 receptor, alpha 1*	NM_001560	-0.037	-0.006	1.732
JJAZ1	*Suppressor of zeste 12 homolog*	NM_015355	-0.416	0.017	-1.499
KIF11	*Kinesin-related mitotic motor protein*	NM_004523	-2.475	-3.832	-2.770
KIF20A	*Kinesin family member 20A*	NM_005733	0.333	0.251	2.845
KIF22	*Kinesin family member 22*	NM_007317	1.007	-0.894	-0.824
KIF23	*Kinesin family member 23*	NM_004856	-0.543	-1.974	0.129
KIF4A	*Kinesin family member 4*	NM_012310	0.232	-0.871	0.901
KPNA2	*Karyopherin (importin) alpha 2*	NM_002266	-0.697	-0.087	1.354
K-RAS	*v-Ki-ras2 Kirsten rat sarcoma viral oncogene homolog*	NM_004985	-0.298	-1.212	1.022
LSM2	*LSM2 homolog, U6 small nuclear RNA associated*	NM_021177	0.259	-0.257	-0.170
LTA	*Lymphotoxin A*	NM_000595	-0.452	-0.194	0.003
MAD2L1	*MAD2 (mitotic arrest deficient, homolog)-like 1*	NM_002358	0.658	-1.459	-0.212
MAP1B	*Microtubule-associated protein 1 B*	NM_005909	-0.307	-1.038	-0.436
MAP4K3	*mitogen-activated protein kinase kinase kinase kinase 3*	NM_003618	-0.451	-1.441	1.760
MCM10	*Minichromosome maintenance deficient 10*	NM_018518	-0.359	-1.539	-1.341
MCM2	*Minichromosome maintenance deficient 2 mitotin*	NM_004526	-0.239	-0.932	1.991
MCM3	*Minichromosome maintenance deficient 3*	NM_002388	-0.518	-1.476	0.412
MCM4	*Minichromosome maintenance deficient 4 homolog*	NM_005914	-0.780	-2.753	-0.600
MCM5	*Minichromosome maintenance deficient 5, cell division cycle 46*	NM_006739	-1.087	-1.563	-0.880
MCM6	*Minichromosome maintenance deficient 6*	NM_005915	-0.593	-0.132	-0.995
MELK	*Maternal embryonic leucine zipper kinase*	NM_014791	0.225	-0.925	-1.274
MSH2	*mutS homolog 2*	NM_000251	1.149	-0.033	-0.417
MSH6	*MutS homolog 6*	NM_000179	1.924	-1.188	0.053
MTBP	*Mdm2, transformed 3T3 cell double minute p53 binding protein*	NM_022045	-0.165	-0.632	-0.470
MYB	*myeloblastosis oncogene*	NM_005375	0.652	-0.779	1.330
NEK2	*NIMA (never in mitosis gene a)-related expressed kinase 2*	NM_002497	-0.176	-0.891	-0.130
NOTCH1	*Notch gene homolog 1*	NM_017617	0.145	-0.554	-1.14
NOTCH2	*Notch gene homolog 2*	NM_024408	1.105	0.0299	0.119
NUCKS	*JC7, Nucks1*	NM_022731	0.166	-0.547	-1.548
PAICS	*Phosphoribosylaminoimidazole carboxylase, phosphoribosylaminoribosylaminoimidazole, succinocarboxamide synthetase*	NM_001079524	0.838	0.186	0.955
PHF6	*PHD finger protein 6*	NM_032335	0.387	-0.730	-0.496
PIK3C3	*phosphoinositide-3-kinase, class 3*	NM_002647	1.094	-0.132	2.345
PIK3R2	*phosphatidylinositol 3-kinase, regulatory subunit, polypeptide 2*	NM_005027	-0.008	-0.350	2.147
PIR51	*RAD51 associated protein 1*	NM_006479	0.102	-1.701	-1.215
PLK4	*Polo-like kinase 4*	NM_014264	-0.354	0.309	0.502
POLA	*Polymerase (DNA directed), alpha 1*	NM_016937	0.791	-0.106	-0.960
PRIM1	*DNA primase, p49 subunit*	NM_000946	1.655	-0.497	-0.012
PRIM2A	*DNA primase, p58 subunit*	NM_000947	0.602	-0.680	-1.481
PSIP1	*PC4 and SFRS1 interacting protein 1*	NM_021144	0.063	-1.017	-0.146
RAD21	*RAD21 homolog*	NM_006265	-0.406	-0.253	0.487

Gene Symbol	Description	Accession #	Z-score MB231	Z-score Hs578T	Z-score MCF10A
RBM3	*RNA binding motif protein 3*	NM_006743	-0.250	-0.182	-0.199
RRM1	*Ribonucleotide reductase M1*	NM_001033	-2.637	-2.053	-0.955
RRM2	*ribonucleotide reductase M2*	NM_001034	-3.647	-2.517	-1.348
S100A6	*S100 calcium binding protein A6*	NM_014624	0.149	-0.341	-0.449
SHCBP1	*Shc binding protein (mPAL)*	NM_024745	0.509	-0.586	0.769
SKP2	*S-phase kinase-associated protein 2 (p45)*	NM_032637	-0.346	-0.437	-0.349
SLBP	*stem-loop binding protein*	NM_006527	-1.094	-0.731	0.306
SMARCA5	*SWI/SNF related, matrix associated, actin dependent regulator of chromatin, subfamily a, member 5*	NM_003601	0.461	-0.783	0.316
SOX11	*SRY-box containing gene 11*	NM_003108	0.654	-0.389	0.854
SPAG5	*S17 protein*	NM_006461	-1.337	-2.613	-0.803
STMN1	*Stathmin 1*	NM_203399	0.126	2.360	0.721
SUV39H1	*Suppressor of variegation 3-9 homolog 1*	NM_003173	-0.958	-1.299	0.221
TACC3	*Transforming, acidic coiled-coil containing protein 3*	NM_006342	1.007	-2.453	0.925
TIMP1	*tissue inhibitor of metalloproteinase 1*	NM_003254	-0.214	-0.927	0.590
TK1	*Thymidine kinase 1*	NM_003258	-0.419	-1.410	-0.637
TOP2A	*Topoisomerase (DNA) II alpha*	NM_001067	-0.654	-0.294	-0.220
TPX2	*TPX2, microtubule-associated protein homolog*	NM_012112	-1.932	-3.864	-1.452
TYMS	*Thymidylate synthase*	NM_001071	-0.157	-1.638	-0.827
UHRF1	*Ubiquitin-like, containing PHD and RING finger domains, 1*	NM_001048201	0.193	-1.696	0.965
UQCRFS1	*Ubiquinol-cytochrome c reductase, Rieske iron-sulfur polypeptide 1*	NM_006003	-1.100	-1.857	-0.526
VEGFC	*vascular endothelial growth factor C*	NM_005429	0.095	-1.015	-0.260
WNT7B	*wingless-related MMTV integration site 7B*	NM_058238	-0.648	-1.064	-0.261
