# Supplementary material for: Cross-species genomic and functional analyses identify a combination therapy using a CHK1 inhibitor and a ribonucleotide reductase inhibitor to treat triple-negative breast cancer
Source: Breast Cancer Res. 2012 Jul 19;14(4):R109. doi: 10.1186/bcr3230 (PMC3680937; doi:10.1186/bcr3230)
Supplement: Additional file 2 — Table S2. Combination Index data for drug synergy in triple negative cells. [file bcr3230-S2.PDF]

Supplementary Table 2

A. Combination Index Data for MDA-MB231		
**Dose GEM (nM)**	**Dose UCN01 (nM)**	**CI**
10.0	1.0	0.264
10.0	5.0	0.097
10.0	10.0	0.138
10.0	50.0	0.211
10.0	100.0	0.301

B. Combination Index Data for M6 cells		
**Dose UCN01 (nM)**	**Dose GEM (nM)**	**CI**
20.0	0.1	0.571
20.0	0.5	0.136
20.0	1.0	0.225
20.0	5.0	0.971

C. Combination Index Data for BT-549 cells		
**Dose GEM (nM)**	**Dose UCN01 (nM)**	**CI**
10.0	5.0	3.339
10.0	10.0	1.114
10.0	50.0	0.563
10.0	100.0	0.215

D. Combination Index Data for SUM159 cells		
**Dose GEM (nM)**	**Dose UCN01 (nM)**	**CI**
4.0	1.0	0.262
4.0	5.0	0.075
4.0	10.0	0.101
4.0	50.0	0.136

E. Combination Index Data for HCC1187 cells		
**Dose GEM (nM)**	**Dose UCN (nM)**	**CI**
10.0	0.5	0.012
10.0	1.0	0.019
10.0	5.0	0.040
10.0	50.0	0.016
