# Supplementary figures and images for: Cross-species genomic and functional analyses identify a combination therapy using a CHK1 inhibitor and a ribonucleotide reductase inhibitor to treat triple-negative breast cancer
Source: Breast Cancer Res. 2012 Jul 19;14(4):R109. doi: 10.1186/bcr3230 (PMC3680937; doi:10.1186/bcr3230)

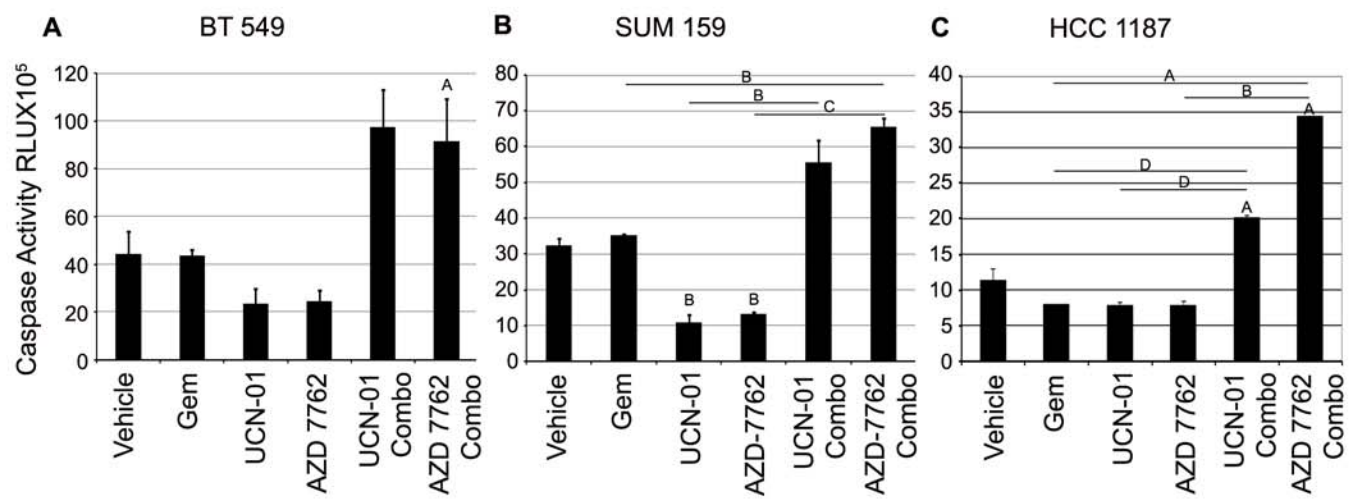

Supplement: Additional file 3 — Figure S1. Combination therapy with Chk1 inhibitors and gemcitabine increases apoptosis in TNBC cells. BT-549 (A), SUM 159 (B) and HCC 1187 (C) cells were treated with agents on day 0 and apoptosis was measured by Caspase 3/7 assay on day two. BT-549 cells (gemcitabine 10 nM; UCN-01 100 nM; AZD 7762 150 nM). SUM 159 (gemcitabine 4 nM; UCN-01 80 nM; AZD 7762 300 nM). HCC 1187 (gemcitabine 10 nM; UCN-01 150 nM; AZD 7762 200 nM). P-value based upon change from vehicle treatment (letters only) and from single agent to combination treatment (line and letter) (A ≤ 0.01, B ≤ 0.005, C ≤ 0.001, D ≤ 0.0005) [file bcr3230-S3.PDF]
